# Zone 0 thoracic branch endoprosthesis with aortic arch debranching

**DOI:** 10.1016/j.jvscit.2026.102524

**Published:** 2026-09-19

**Authors:** Philip Skummer, Paul Dyrud, Brandon Key, Michael Malinowski

**Affiliations:** a Division of Vascular and Interventional Radiology, Department of Radiology, Medical College of Wisconsin, Milwaukee, WI; b Division of Vascular and Endovascular Surgery, Department of Surgery, Medical College of Wisconsin, Milwaukee, WI

**Keywords:** Aortic arch repair, Zone 0 endovascular repair, Aortic arch debranching, Hybrid open and endovascular aortic aneurysm repair

## Abstract

We present a case of staged arch debranching with subsequent zone 0 branched thoracic aortic endograft in a patient with a bovine arch. Open surgery is the historic gold standard for aortic arch repair. However, poor operative candidates may be unable to tolerate sternotomy and circulatory arrest required for repair. This case highlights a staged hybrid endovascular and open approach to repairing a progressively aneurysmal aortic arch.

Pathology requiring aortic arch repair can include progressive aneurysmal enlargement, dissection, pseudoaneurysm at site of prior open repair, intramural hematoma, and penetrating atherosclerotic ulcer (PAU).^1^, ^2^, ^3^ Open surgical repair is the gold standard for aortic arch pathology; however, there is significant 30-day mortality between 5% and 10% associated with open repair.^1^^,^^4^, ^5^, ^6^ Occasionally, the left subclavian artery (LSA) can be covered to allow for aortic arch repair from a purely endovascular approach; however, revascularization of the carotid arteries is mandatory for intracranial perfusion. A shared common origin of the brachiocephalic and left common carotid arteries (LCCA) (bovine arch) is one of the most common anatomic variations of the aortic arch. Alternatives to open repair of the aortic arch include both hybrid and endovascular approaches to maintain intracranial perfusion, such as physician-modified endografts, in situ or customized fenestrated/scalloped endografts, periscope/snorkel endografts, or branched endografts.^1^, ^2^, ^3^, ^4^^,^^7^, ^8^, ^9^, ^10^, ^11^ Zone 0 placement of the endograft may be required for adequate proximal seal in patients with a bovine arch.^7^^,^^8^ This case highlights a hybrid operating approach to maintain perfusion to all major aortic branch vessels in a patient with bovine arch anatomy who was unable to undergo open aortic repair of a progressively enlarging PAU. Informed consent was obtained from the patient for the publication of this report and accompanying images.

## Case description

We present a case of a 77-year-old man with slowly enlarging aortic arch PAU who is not a robust surgical candidate given poor functional capacity (metabolic equivalent <4) and American Society of Anesthesiologists class 4 due to extensive past medical history. Notable patient history includes heart failure with reduced ejection fraction (41%-45%), grade 2 cardiac diastolic dysfunction, moderate mitral valve regurgitation, mild tricuspid valve regurgitation, moderate aortic valve regurgitation, mild pulmonary hypertension, type 2 diabetes (hemoglobin A1c 6.7% on metformin), prior transient ischemic attack, and primary hypertension.

The patient’s PAU was first imaged approximately 4 years before the index operation with a depth of 20 mm, a width of 40 mm, and a total aortic diameter of 55 mm. The PAU is centered at the origin of the LSA extending into zone 2 and zone 3 (Fig 1). The patient has variant anatomy with shared origin of the brachiocephalic artery and LCCA (bovine arch). After undergoing multidisciplinary review, in coordination with the patients’ desire to forego treatment at that time based on the review of possible operative complications, the decision was made for continued surveillance in early discussions. Although there are no widely accepted consensus guidelines to guide the management of PAU in this location of the arch, recent literature shows growing expert consensus for descending thoracic PAU/intramural hematoma. A review in *EJVES* in 2025 exhibited ≥80% expert consensus on 18 of 25 clinical recommendation guideline statements and 7 statements achieving full expert consensus.^12^ However, this location of the arch lacks similar guiding statements. Our practice is to offer repair for patients deemed symptomatic or with total aortic diameter exceeding 55 mm or 60 mm. Ultimately, there was progressive growth to 32-mm depth, 49-mm width, and 70-mm total aortic diameter; the proximal edge of the aneurysmal thoracic aorta/PAU is within 12 mm of zone 0, thus requiring stent graft extension into zone 0 for adequate proximal seal. As surgical discussions progressed, the patient exhibited a desire to undergo repair based on the annual rupture risk associated with his larger arch diameter during follow-up. He was offered nonoperative care as one of many options, but he and his family felt that he maintained a suitable quality of life that warranted aneurysm repair for long-term survival. Based on his other comorbidities, cardiac surgery deemed the patient too high risk for open arch reconstruction.

**Fig 1:**
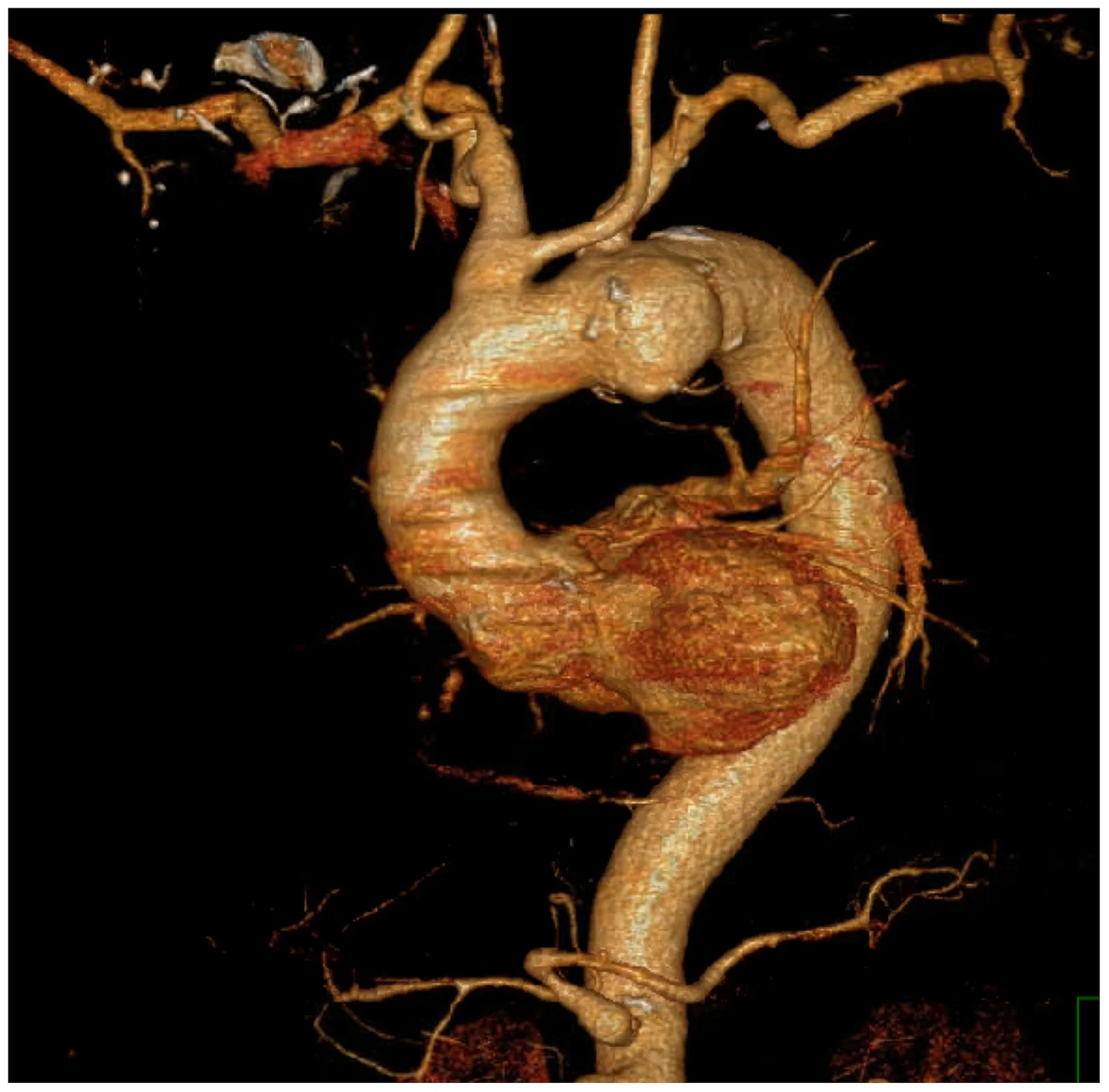
Preoperative computed tomography three-dimensional reconstruction of the aorta demonstrates bovine arch anatomy with large penetrating atherosclerotic ulcer (PAU) just distal to the innominate artery.

The patient was preadmitted to the intensive care unit (ICU) for lumbar drain placement before staged aortic arch debranching with a subsequent zone 0 thoracic branch endograft (TBE) during the same hospital admission. Our newer protocol for spinal cord ischemia rescue has since progressed at our intuition with more expeditious drain placement such that this would not be required currently for a similar case within our practice. His initial operation consisted of a retroesophageal right common carotid artery to LCCA bypass using an 8-mm ringed polytetrafluoroethylene bypass graft, an LCCA to subclavian artery bypass using an 8-mm ringed polytetrafluoroethylene graft with proximal LCCA ligation and distal transposition to the bypass, and coil embolization of the LSA from a left transfemoral approach as well as angiographic evaluation of the bypass construct. The estimated blood loss for this portion of the operation was 100 mL with a reference air kerma of 2118 mGy and a total contrast volume of 100 mL of Omnipaque300 (GE Healthcare). The patient returned to the ICU without any change in neurovascular examination.

On postoperative day 1, he returned to the operating room for zone 0 TBE using a 45 × 150-mm GORE TBE with a 12-mm portal. The side branch from the endograft was extended distal to the ligated origin of the LCCA into the brachiocephalic artery using overlapping 20 × 60-mm GORE endografts, and the main body was extended using a 45 × 45 × 100-mm GORE TAG conformable thoracic endograft into the distal zone 4 of the thoracic aorta (W.L. Gore & Associates, Inc) (Fig 2). The main body of the graft was delivered through a 26F sheath in the right common femoral artery with a through-and-through wire from the right radial artery that had a 6F slender sheath. Flush catheter for angiography was delivered through a left common femoral artery 5F sheath. No venous access was obtained for balloon occlusion of the inferior vena cava/right atrium for preload reduction and cardiac output suppression; however, permissive hypotension with systolic blood pressure less than 100 mm Hg was obtained before deployment of the branched endograft main body. Incidental note was made of nonopacification of the left vertebral artery; however, it was unclear whether this was retrograde flow due to a change in flow dynamics resulting from debranching or an acute occlusion. Otherwise, no intraprocedure complications were noted including retrograde aortic dissection or valve injury, which was confirmed by transesophageal echocardiography performed by cardiac anesthesiology. The estimated blood loss for this portion of the operation was 50 mL with a reference air kerma of 1303 mGy and a total contrast volume of 220 mL of Omnipaque 300.

**Fig 2:**
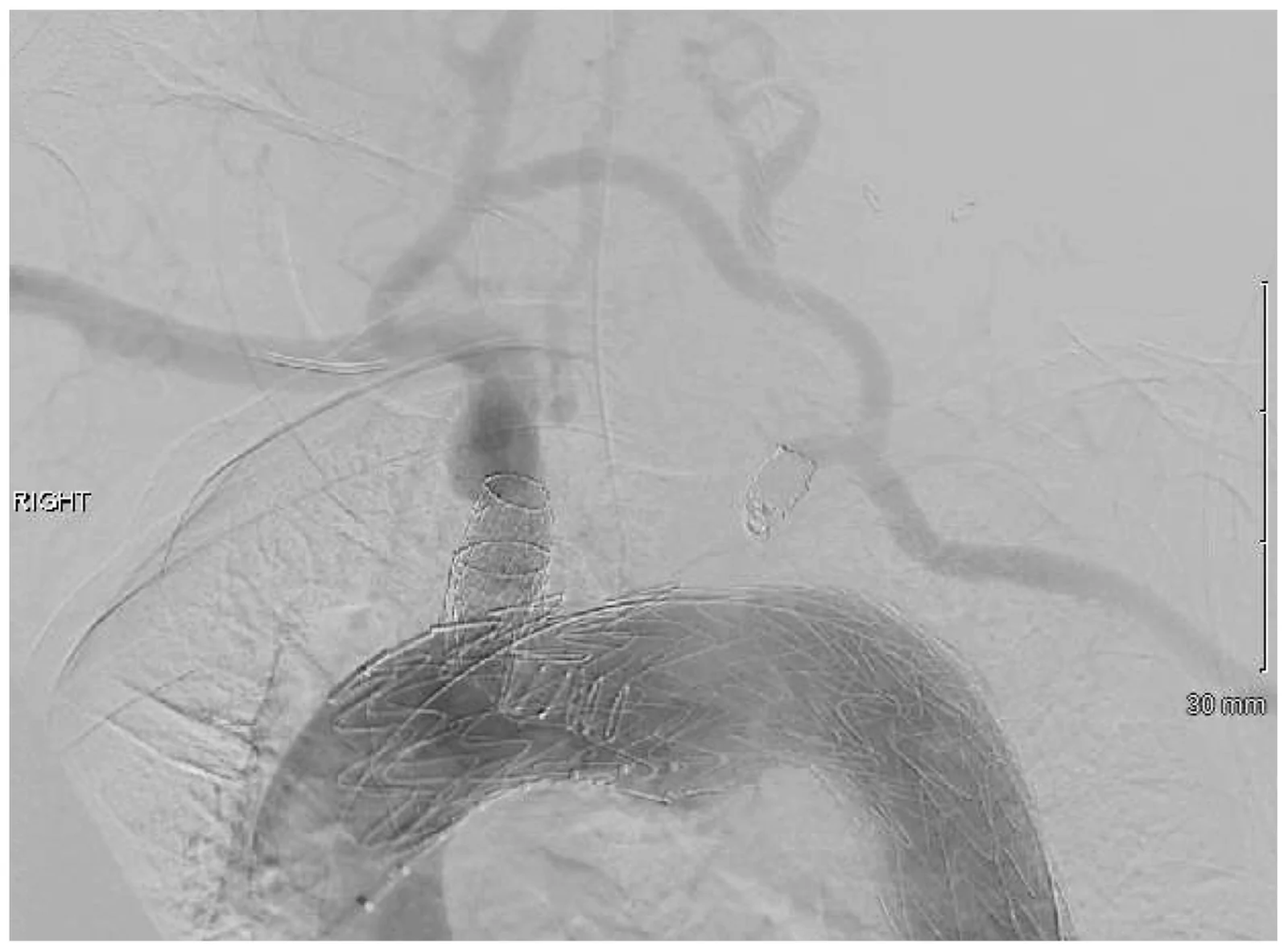
Intraoperative imaging demonstrates aortic arch debranching with a right common carotid to left subclavian artery (LSA) bypass with the transposed left common carotid artery (LCCA) sewn to the bypass graft. In addition, this image demonstrates a zone 0 thoracic branch endoprosthesis with a branch into the innominate artery and a coil-embolized proximal LSA.

The patient returned to the ICU in stable condition without focal neurologic deficit. Given the lack of symptoms, nonopacification of the left vertebral artery was not further investigated during the hospital admission. The patient remained in the ICU until postoperative day 3 and was started on dual antiplatelets (aspirin 81 mg and clopidogrel 75 mg). He was ultimately discharged home on day 6 after clearance by physical and occupational therapy. Follow-up computed tomography angiography of the neck and chest was performed approximately 2 months after discharge, demonstrating an appropriately positioned endograft with patent carotid-carotid-subclavian bypasses (Fig 3). The left vertebral artery is patent, and previous angiographic findings were presumably related to retrograde flow. At 2 months, the aortic dimensions were not significantly changed, and there was no evidence of endoleak. The patient was at his baseline state of health without a change in neurovascular status, wound complications, or dysphagia. The aneurysm has continued to regress to 6.7 cm on computed tomography angiography on his 11-month postoperative scan, with no evidence of endoleak, component migration, or conduit occlusions, and the patient was in good health at a 14-month in-person clinic follow-up postoperatively.

**Fig 3:**
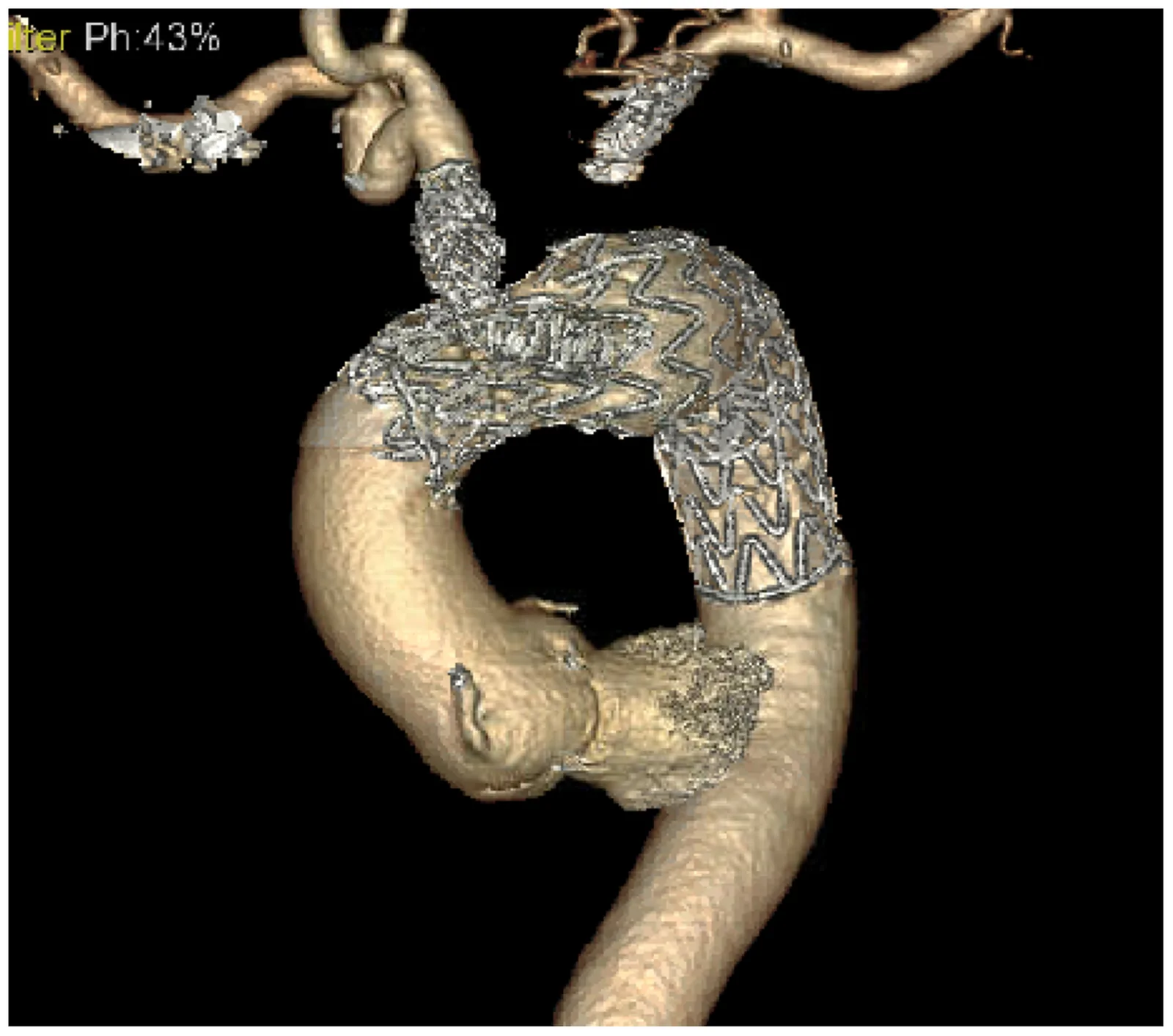
Early interval postoperative computed tomography three-dimensional reconstruction demonstrating aortic arch repair with a zone 0 branched endograft and aortic arch debranching.

## Discussion

Traditionally, endovascular repair of the thoracic aorta was limited to zone 3 and distal; more devices are coming on the market to address aortic arch pathology,^3^^,^^6^ thus leading to changes in the treatment paradigm.^4^ There is a growing body of evidence for zone 0 repairs with off-label use of different endografts,^10^^,^^13^^,^^14^ much of which has come from case reports and small case series. Stent grafting of zone 0 has high technical success with multiple devices.^1^, ^2^, ^3^, ^4^^,^^7^^,^^8^ There is a high risk of complication with zone 0 instrumentation compared with other aortic zones, including stroke and 30-day mortality;^2^^,^^10^ however, these hybrid approaches result in overall lower mortality compared with open aortic arch repair.^4^ There is no currently agreed on technique for addressing aortic arch pathology from a hybrid approach; therefore, such repairs should be performed at high-volume centers with multidisciplinary discussion to involve vascular surgery, interventional radiology, and cardiothoracic surgery.^1^ Midterm patency and durability of TBE devices,^6^ such as the one used in this patient, have encouraging results. Further research is warranted to confirm durability of zone 0 repairs, patient selection, and optimal medical management, such as antiplatelet/anticoagulation, after placement of TBE devices.

The current case highlights an unusual bovine arch anatomy with early LCCA takeoff, treated with arch debranching requiring a right common carotid to left subclavian bypass, with left common carotid transposition onto this conduit and proximal LSA embolization. This hybrid approach then allowed for staged zone 0 TBE deployment with appropriate seal zones and durable reconstruction. The other options available would have been challenging with an open arch reconstruction in a high-risk surgical patient and ultimately limited by the proximal left common carotid origination for any other endovascular techniques including physician-modified endograft, branch, or fenestrated repair due to short proximal innominate lengths.

## Conclusions

Staged aortic arch debranching with a subsequent zone 0 branched thoracic aortic endograft in a patient with bovine arch anatomy demonstrates short- and midterm clinical success for treatment of progressively enlarging PAU.

## Funding

None.

## Disclosures

None.

## References

[1] Rockley M., Rommens K.L., McClure R.S., Herget E.J., Smith H.N., Moore R.D. (2023). Aortic arch endovascular branch and fenestrated repair: initial Canadian experience with novel technology. J Vasc Surg Cases Innov Tech.

[2] Nana P., Spanos K., Panuccio G. (2024). Branched and fenestrated endovascular aortic arch repair in patients with native proximal aortic landing zone. J Vasc Surg.

[3] Dake M.D., Bavaria J.E., Singh M.J. (2021). Management of arch aneurysms with a single-branch thoracic endograft in zone 0. JTCVS Tech.

[4] Kudo T., Kuratani T., Shirakawa Y. (2022). Effectiveness of proximal landing zones 0, 1, and 2 hybrid thoracic endovascular aortic repair: a single centre 12 year experience. Eur J Vasc Endovasc Surg.

[5] Tanaka Y., Mikamo A., Suzuki R. (2014). Mortality and morbidity after total aortic arch replacement. Ann Thorac Surg.

[6] Liang N.L., Dake M.D., Fischbein M.P. (2022). Midterm outcomes of endovascular repair of aortic arch aneurysms with the gore thoracic branch endoprosthesis. Eur J Vasc Endovasc Surg.

[7] Toya N., Ohki T., Fukushima S., Shukuzawa K., Ito E., Akiba T. (2018). Fenestrated endovascular repair of aortic arch aneurysm in patients with bovine arch using the Najuta stent graft. J Vasc Surg Cases Innov Tech.

[8] Toya N., Ohki T., Fukushima S. (2018). Case series of aortic arch aneurysm in patients with bovine arch treated with proximal scalloped and fenestrated stent graft. Cardiovasc Intervent Radiol.

[9] Bedeir K., Chad McRee C., Johnson W.E. (2023). Concomitant carotid-carotid-axillary bypass with zone 0 branched endograft and transcatheter aortic valve-in-valve replacement via retroperitoneal access. JTCVS Tech.

[10] Zhu L., Li X., Lu Q. (2023). A systematic review and meta-analysis of thoracic endovascular aortic repair with the proximal landing zone 0. Front Cardiovasc Med.

[11] Arima D., Suematsu Y., Nishi S., Kurahashi K., Shimizu T., Yoshimoto A. (2020). Zone 0 thoracic endovascular aortic repair using reverse extra-anatomical aortic arch debranching technique for an anastomotic pseudoaneurysm and acute aortic dissection that developed after Bentall’s surgery combined with Sjogren’s syndrome. Ann Vasc Dis.

[12] Squizzato F., D'Oria M., Antonello M. (2026). International expert consensus on the management of acute aortic type B intramural haematoma and penetrating ulcer. Eur J Vasc Endovasc Surg.

[13] Marrocco-Trischitta M.M., Alaidroos M., Romarowski R.M. (2020). Geometric pattern of proximal landing zones for thoracic endovascular aortic repair in the bovine arch variant. Eur J Vasc Endovasc Surg.

[14] Elbayoumi E.H., Farres H., Polania-Sandoval C.A., Lanka S.P., Erben Y. (2024). Complex zone 0 aortic arch repair using TBE device: case report and literature review. Ann Vasc Surg Br Rep Innov.

